# Cord blood antimicrobial peptide LL37 levels in preterm neonates and association with preterm complications

**DOI:** 10.1186/s13052-022-01295-6

**Published:** 2022-07-08

**Authors:** Zhuxiao Ren, Wenhui Mo, Liling Yang, Jianlan Wang, Qi Zhang, Zhicheng Zhong, Wei Wei, Zhipeng Liu, Zhiping Wu, Yao Yao, Jie Yang

**Affiliations:** 1 Department of Neonatology, Guangdong Women and Children Hospital, Guangzhou, China; 2 Department of Neonatology, Foshan fosun chancheng hospital, Foshan, China; 3 Clinical Genetic Center, Guangdong Women and Children Hospital, Guangzhou Medical University, Guangzhou, Guangdong China; 4 Guangdong Cord Blood Bank, Guangzhou, China; 5 Guangdong Provincial Key Laboratory of Stem Cell and Regenerative Medicine, Center for Cell Regeneration and Biological Therapies, Guangzhou Institutes of Biomedicine and Health, Chinese Academy of Sciences, Guangzhou, China; 6 Department of Neonatology, Nanfang Hospital, Southern Medical University, Guangzhou, China

**Keywords:** Antimicrobial peptide, LL37, Preterm neonates, Preterm complications

## Abstract

**Background:**

Cathelicidin/LL-37 plays a significant role in the human immune defense reaction. Preterm human immature organs being exposed to inflammation-induced injury was the critical denominator leading to the common preterm associated complications. Previous study showed LL37 concentration in preterm neonates was lower in tracheal aspirates and breast milk as compared to term infants. An adults study showed decreased LL-37 levels was a risk factor for patients in developing severe chronic obstructive pulmonary disease (COPD). However, little is known about the regulation of human cord blood LL37 in preterm neonates and the association with preterm complications. This study was designed to investigate the concentration of LL37 in cord blood of preterm infants and correlation with preterm complications.

**Methods:**

Singleton infants born in June 2017 to August 2021 in the study hospital were enrolled. Maternal and neonatal clinical characteristics were collected. LL37 levels, pro-inflammatory factor interleukin-6 (IL-6) and tumor necrosis factor-a (TNF-a) in cord blood and LL37 levels in serum 48–72 hours after birth were measured by enzyme-linked immunosorbent assay. The serum level of LL37 in preterm and term neonates were compared, the perinatal factors possibly affecting the LL37 levels were investigated and the relationship between LL37 level and preterm outcomes were analyzed.

**Results:**

Cord blood LL37 levels in preterm infants were lower than that in term neonates. Cord blood LL37 level was positively correlated with gestational age in preterm. Prenatal steroid administration in preterm neonates decreased cord blood LL37 level. LL37 level was obviously lower in patients with bronchopulmonary dysplasia (BPD). Multiple line regression analysis showed higher LL37 level in cord blood was an independent protective factor for BPD. The concentration of pro-inflammatory factor IL-6 was negatively correlated with LL37.

**Conclusion:**

Cord blood LL37 levels increased during gestation and decreased after perinatal steroid usage. Very preterm infants who displayed higher cord blood LL37 level had reduced risk of developing BPD. Regulation of pro-inflammatory cytokine IL-6 may be associated with the protective effect of LL37 on BPD.

**Supplementary Information:**

The online version contains supplementary material available at 10.1186/s13052-022-01295-6.

## Background

Cathelicidin/LL-37, the only known human cathelicidin, belongs to the most important human antimicrobial peptides families and plays a significant role in the human immune defense reaction, including regulation of innate immunity and inflammation-induced tissue repair [1–7]. The role of LL37 in inflammation associated diseases is controversial. Previous studies in adults showed decreased LL-37 levels could be high risk factor for patients in developing severe chronic obstructive pulmonary disease (COPD) [8], but also contributed to arthritis [9]. Exposure to inflammatory mechanisms constitutes the cornerstone of the complications associated with prematurity [10–12]. Among the preterm complications, bronchopulmonary dysplasia (BPD) is still the primary major complication of preterm birth causing neonatal mortality and morbidity [10–12]. Persistent prenatal and postnatal inflammation plays a crucial role in the pathogenesis of bronchopulmonary dysplasia (BPD) [12]. In adult COPD study, it was found the lower LL37 lever was associated with higher NF-κB activation, therefore contributing to chronic inflammatory response [8]. Previous study showed a significant correlation was observed between maternal and cord plasma LL37 levels [13]. LL37 concentration in preterm infants was lower in tracheal aspirates and breast milk as compared to term infants [14, 15], and LL37 level was elevated in tracheal aspirates during respiratory infection [15]. However, little is known about human cord blood LL37 in preterm neonates and the association with preterm complications, as well as the underlying mechanisms. This study was designed to investigate the concentration of LL37 in cord blood of preterm infants and the correlation with the development of preterm complications.

## Methods

### Patients enrollment

This was a prospective cohort study. Singleton infants born from June 2017 to August 2021 in Guangdong Women and Children Hospital were included in this study. Inclusion criteria: (a) newborns born in the study hospital; (b) singleton birth; (c) newborns guardian’ written consent was obtained. Exclusion criteria: (a) with major congenital abnormalities; (b) the mother was positive for hepatitis B (HBsAg and/or HBeAg) and C virus (anti-HCV), syphilis, Human immunodeficiency virus (HIV) (anti-HIV-1 and anti-HIV-2) and IgM against cytomegalovirus, rubella, toxoplasma, and herpes simplex virus or mother infection, such as chorioamnionitis and sepsis (chorioamnionitis, TORCH (toxoplasma, rubella virus, cytomegalovirus, herpes simplex virus, and other) syndrome was diagnosed [16].; (c) severe perinatal asphyxia (defined as an Apgar score of 0–3 for more than 5 minutes, a cord blood gas pH < 7.00, or both) [17].

### Clinical data collection

Maternal clinical information included age, gestational diabetes mellitus, pregnancy-induced hypertension, premature rupture of membrane and antenatal steroids administration.

Neonatal clinical data included gender, gestational age, birth weight, delivery mode and mild asphyxia.

Bronchopulmonary dysplasia (BPD) was defined as treatment with oxygen > 21% for at least 28 days by using the diagnostic criteria proposed in 2001 by the Eunice Kennedy Shriver National Institute of Child Health and Human Development (NICHD, Bethesda, MD). Its severity will be assessed at 36 weeks of postmenstrual age or discharge home whichever comes first. Mild BPD, defined as breathing room air at assessment. Moderate BPD, defined as the need for < 30% supplemental oxygen, and severe BPD defined as needing ≥30% supplemental oxygen or positive airway pressure. Cranial ultrasonography was recommended during the first week, between the 14th and 28th days of life, and between 34 and 36 weeks of postmenstrual age if the infant was still hospitalized in the study center at that time. Necrotizing enterocolitis (NEC) was diagnosed during surgery, at autopsy, or by a finding of pneumatosis intestinalis, hepatobiliary gas, or free intraperitoneal air on radiography [18]. All stages of retinopathy of preterm (ROP) were recorded according to the international classification. Culture-positive sepsis was defined by a positive blood or cerebral spinal fluid culture. All clinical diagnoses were defined according to a standard reference [17].

### Cord blood collection and LL37 concentration detection

Cord blood and abandoned blood from routine blood tests 48–72 hours after birth were collected and then centrifuged in 4 degree celsius with gravity of 500 g for 8 minutes in low-temperature centrifuge (RT, Beckman, USA). Serum from cord blood was separated for assessment of LL37 (ng/ml) and IL6, TNF-a (pg/ml) concentration. Blood samples were collected only when it was clinically required during routine care. The minimum volume of serum for enzyme-linked immunosorbent assay (ELISA) kit (LL-37 ELISA kit, elabscience, E-ELH2438c, 6KMWT3F5HA; Human IL-6/Interleukin-6 ELISA Kit, Wuhan EIAab Science, Wuhan China, Cat.E0079h; Human TNF-a/ Tumor necrosis factor a ELISA Kit, Wuhan EIAab Science, Wuhan China, Cat.E0133h) was 5 ul.

### Statistical analysis

Means and standard deviation are reported for continuous variables, and the number and percentage are reported for categorical variables. Group comparisons of categorical variables were performed using nonparametric tests, the Fisher’s exact test, or chi-square test, as appropriate. Differences in continuous values were compared using an unpaired Student’s t-test between two groups. Pearson/Spearman’s correlation were used to determine associations between LL37 levels and variables. Multiple linear regression analysis was used to estimate the predictive contribution of neonatal/maternal factors on LL37 level or the contribution of factors on BPD. The variables’ distribution characteristics were estimated with single sample Kolmogrov-Smirnov test. All statistical tests were two-tailed, and *p*-values < 0.05 were deemed statistically significant. All statistical analysis were done using SPSS 21.0 (IBM).

## Results

### Study population

A total of 245 infants were enrolled in this study between June 2017 and August 2021. Eighty (29.8%) were term neonates and 165 (70.2%) were preterm neonates, among which 112 were very preterm neonates less than 32 gestational weeks (GA). There were significant differences in gestational age, birth weight, delivery mode, maternal age, maternal pregnancy-induced complication and antenatal steroids use between term and preterm neonates (Table 1).

**Table 1:** Infants and maternal characteristics

Characteristic	Term(*n* = 80)	Preterm(*n* = 165)	*P* value
GA (Weeks)	39.5 ± 1.0	31.5 ± 2.7	0.000
Birth weight (kilogram)	3.3 ± 0.4	1.6 ± 0.5	0.000
Gender (male, %)	52.5	55.2	0.769
Cesarean section delivery (%)	33.8	52.7	0.012
Mild asphyxia (%)	15	12.5	1.0
Maternal age (year)	29.3 ± 3.8	32.2 ± 6.3	0.000
GDM (%)	12.5	29.9	0.008
Pregnancy-induced hypertension (%)	0	13.4	0.000
Antenatal steroids usage (%)	2.5	56.4	0.000

*GA* gestational age, *GDM* gestational diabetes mellitus

### Cord blood and serum LL37 level

**Fig. 1:**
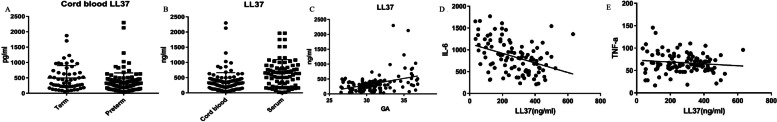
**A** Cord blood LL37 level in preterm and term neonates (mean and standard deviation (SD):term neonates-497.19 ± 404.98 vs preterm infants-355.21 ± 308.79, *P* = 0.01). **B** Cord blood and serum LL37 level in preterm neonates (mean and SD: cord blood-355.21 ± 308.79 vs serum 48–72 hours after birth-644.87 ± 449.32, *P* = 0.01). **C** Correlation between LL37 and gestation age in preterm infants (R = 0.347, *P*<0.0001, *n* = 134). **D** Correlations between IL-6 and LL37 level in very preterm infants (R = -0.391, *p*<0.0001, *n* = 102). **E** Correlations between TNF-a and LL37 level in very preterm infants (R = 0.120, *p* = 0.230, *n* = 102)

Fifty-five term and 134 preterm infants (102 very preterm infants less than 32 GA) enrolled had available cord blood LL37 levels. Cord blood LL37 levels were significantly lower in preterm neonates than term neonates (*P* = 0.01, Fig. 1A). Among 134 preterm infants with available cord blood LL37 levels, 75 infants had available serum LL37 levels 48–72 hours after birth. Serum LL37 levels 48–72 hours after birth in preterm were higher than that in cord blood (*P* = 0.01, *n* = 75, Fig. 1B). Further, we investigated possible factors that may affect the concentration of LL37 in cord blood of preterm infants. Multiple regression analysis showed antenatal steroid use could reduce the cord blood levels of LL37 (B = -126.695, *p* = 0.027, *n* = 134, Table 2). Serum LL37 level 48–72 hours after birth in preterm infants was not affected by the perinatal factors significantly (Supplemental Table 1, *n* = 75). Lower GA was associated with lower LL37 level in cord blood (B = 45.560, *p* = 0.004, *n* = 134, Table 2). A further linear correlation analysis showed cord blood levels of LL37 were positively correlated with gestation age (*R* = 0.347, *P*<0.0001, *n* = 134, Fig. 1C).

**Table 2:** Multiple regression analysis model for perinatal factors that may affect the concentration of LL37 in cord blood (*n* = 134)

Variables	B	95% CI for B	P
GA (weeks)	45.560	14.985, 76.135	***0.004***
Male	−32.289	−141.544, 76.967	0.559
Birth weight (kilogram)	−17.479	− 194.275, 159.317	0.845
Cesarean section delivery	30.522	−83.053, 144.096	0.595
Pregnancy-induced hypertension	117.669	−57.925, 293.263	0.187
GDM	102.260	−20.012, 224.532	0.100
Antenatal steroids usage	−126.695	− 238.351, -15.039	***0.027***
Premature rupture of membrane	−6.703	− 207.165,193.760	0.947
Maternal age	−7.684	−17.710,2.343	0.132

B: unstandardized regression coefficient. Dependent variable: cord blood LL37 levels

*CI* confidence interval, *GA* gestational age, *GDM* gestational diabetes mellitus

### Cord blood levels of LL37and very preterm infants outcomes

The main characteristics and preterm complications of very preterm infants were showed in Supplemental Table 2. To investigate the associations between LL37 levels and very preterm infants outcomes, we compared the levels of LL37 in very preterm groups between who were diagnosed with the following common complications including BPD, NEC, intraventricular hemorrhage (IVH), ROP, sepsis and these who were not. We found the cord blood levels of LL37 in neonates who later developed BPD were lower than these without BPD (*p* = 0.001). The difference of LL37 in other complications did not reach statistically significance (*n* = 102, Table 3).

**Table 3:** Cord blood levels of LL37 and preterm complications

Complications	Cord blood LL37		P
	Yes	No	
BPD(n)	203.9 ± 112.4 (27)	299.3 ± 131.3 (75)	***0.001***
IVH(n)	258.6 ± 113.3 (37)	282.9 ± 143.0 (65)	0.377
ROP(n)	298.1 ± 86.1 (18)	268.9 ± 140.8 (84)	0.399
NEC(n)	259.1 ± 131.5 (17)	277.0 ± 133.8 (85)	0.624
Sepsis(n)	317.6 ± 98.5 (13)	267.7 ± 136.5 (89)	0.207

*BPD* bronchopulmonary dysplasia, *IVH* intraventricular hemorrhage, *ROP* retinopathy of preterm, *NEC* necrotizing enterocolitis

### Cord blood levels of LL37 and BPD in very preterm neonates

To further investigate the protective contribution of cord blood LL37 and BPD in very preterm infants, we used multiple regression analysis which included perinatal factors that may affect the incidence of BPD. The results showed higher level of LL37 was an independent protective factor of BPD (*n* = 102, Table 4). Further, we also performed multiple regression analysis and tested the association between cord blood LL37 and BPD severity (no BPD, mild, moderate and severe BPD) assessed at 36 weeks gestational age or discharge home whichever comes first. We found sepsis was a risk factor for severer BPD, infants with larger GA and higher cord blood LL37 had less risk to develop severer BPD (Table 5).

**Table 4:** Multiple regression analysis model for factors that may contribute to BPD

Variables	B	95% CI for B	P
Male	0.069	−1.464,0.254	0.459
GA (weeks)	−0.024	−0.117,0.050	0.512
Pregnancy-induced hypertension	0.148	−0.099,0.420	0.279
GDM	−0.161	− 0.125,0.048	0.127
Antenatal steroids usage	0.107	−0.369,0.313	0.298
Cord blood LL37	−0.001	−0.099,0.000	***0.013***
Serum LL37	0.000	−0.002,0.000	0.367
Sepsis	−0.015	0.000,0.407	0.943
Respiratory support duration (days)	0.011	−0.436,0.016	***0.000***
PDA	0.115	0.006,0.353	0.330

B: unstandardized regression coefficient

Dependent variable: BPD

*CI* confidence interval, *GA* gestational age, *PDA* patent ductus arteriosus, (pharmacologically and/or surgically treated)

*GDM* gestational diabetes mellitus

**Table 5:** Multiple regression analysis model for factors that may contribute to BPD severity

Variables	B	95% CI for B	P
Male	0.106	−0.202,0.414	0.497
GA (weeks)	−0.124	−0.234,-0.014	***0.028***
Pregnancy-induced hypertension	0.099	−0.321,0.520	0.640
GDM	0.129	−0.215,0.472	0.459
Antenatal steroids usage	0.085	−0.232,0.403	0.595
Cord blood LL37	−0.002	−0.003,-0.001	***0.003***
Sepsis	0.752	0.262,1.243	***0.003***
PDA	0.134	−0.188,0.456	0.411

B: unstandardized regression coefficient

Dependent variable: BPD severity (no BPD = 0, mild BPD = 1, moderate BPD = 2, and severe BPD = 3)

*CI* confidence interval, *GA* gestational age, *PDA* patent ductus arteriosus, (pharmacologically and/or surgically treated)

*GDM* gestational diabetes mellitus

### Cord blood levels of LL37 and inflammatory cytokines

Perinatal inflammation plays a crucial role in the pathogenesis of bronchopulmonary dysplasia. The pro-inflammatory process affected the undeveloped preterm lungs. We therefore analyzed the correlation between cord blood LL-37 and the common pro-inflammatory cytokines contributing to development of BPD-IL-6 and TNF-a. 102 very preterm infants had available LL37, IL-6 and TNF-a level results. We found IL-6 level was conversely correlated with LL37 level (*R* = -0.391, *p*<0.0001, *n* = 102, Fig. 1D). There were no significant correlations between TNF-a and LL37 level (*R* = 0.120, *p* = 0.230, *n* = 102, Fig. 1E).

## Discussion

Preterm neonates were often exposed to insults like pulmonary infection, invasive mechanical ventilation and hyperoxia, which causes aberrant regulation of lung inflammation [19–22]. Antimicrobial peptides LL37 was the only known human cathelicidin [1–3]. It was an effector molecule of the inflammatory response system of the lung [23]. This current study firstly investigated the cord blood level of LL37 in preterm neonates and the association with preterm complications. The results showed (1) Cord blood LL37 level increased during gestation; (2) Prenatal steroid usage reduced cord blood LL37; (3) LL37 was an independent protective factor for developing BPD. Very preterm infants with higher cord blood LL37 also showed less risk to develop severer BPD, the protection may be associated with an regulating effect on inflammatory response.

In human beings, the cathelicidin LL-37 was regarded as the major microbicidal peptides, as it provided a first line of defense against infection and played a vital role in innate immunity regulation in newborn infants, especially in preterm neonates [4, 24, 25]. The level of LL-37 in neonates was evaluated in several previous studies [1–5, 13–15]. Previous study showed the average LL37 level in neutrophils derived from cord blood of full-term newborns has been demonstrated to be much lower than in adult blood [4]. Susan Schaller demonstrated preterm infants exhibited reduced LL37 in tracheal aspirates as compared to term infants, and an up-regulation of bronchoalveolar lavage fluid LL37 in response to infection probably represented an effector molecule of the respiratory defense system [15]. G. Velázquez-Sámano showed increased LL-37 in the sputum was associated with less respiratory infections [26]. However, less investigation was conducted in preterm neonates among whom the LL37 played a more important role in their less mature immunity system [3, 4, 27]. Our study firstly showed cord blood LL37 level increased during gestation. The LL37 levels in preterm infants were significantly lower than term babies. Considering the immunomodulation and antimicrobial effect of LL37 [1–4, 13–15], the lower LL37 may contribute to higher susceptibility to sepsis and excessive inflammatory response in preterm neonates. And its increasing can be considered as a response to the acute inflammatory or infectious insults.

In the further multiple regression analysis investigating the factors affecting LL37 level, we found antenatal steroid use could reduce the cord blood level of LL37, but did not affect the serum LL37 level after 48–72 hours. The serum LL37 level after 48–72 hours was higher than that in the cord blood. Previous study showed LL37 could be induced after LPS exposure [5]. The increased of LL37 level later after birth might be attributed to regulation by pro-inflammatory extrauterine micro-environment and acted as a favorable feedback for preterm neonates.

Inflammation induced injury were regarded to be the common denominator in all these multi-factorial origins resulting in preterm complications [6–8]. Neonatal immature immune system, particularly in preterm infants, showed a stronger pro-inflammatory signature after injurious exposure [27, 28]. A study in adult showed decreased LL-37 levels could be high risk for patients in develop severe COPD which disease was also characterized as chronic lung inflammation [6]. The underlying mechanism might be that LL37 could inhibit nuclear factor-kappa B thus alleviating expression of inflammatory cytokines networks. In other studies of adults, low LL-37 was found to be associated with poor prognosis in several conditions including chronic dialysis, sepsis, and tuberculosis [29–31]. However, this may not the same condition in neonates because of the difference of immunity system compared with adults. No research was ever conducted in BPD patients yet. Since LL37 level reduced as gestational age (GA) decreased and very preterm infants were at higher risk for preterm associated complications, we compared the cord LL37 level in different complications to access the LL37 correlation with preterm outcomes. We found these very preterm neonates later developed BPD had lower cord blood LL37, and multiple regression analysis showed higher LL37 is an independent protective factor for developing BPD as well as severer BPD. Compared with children with mild or moderate BPD, those with severe BPD had more in-hospital mortality, poorer prognosis of respiratory system and long-term neurodevelopment outcome [32]. This indicated LL37 could promote preterm pulmonary development, which laid foundation for translational application of LL37 in preventing preterm BPD.

The association between inflammatory responses and aberrant lung development have been well established [10, 19]. Inflammatory mediators, which are increased during lung injurious processes after invasive mechanical ventilation, exposure to hyperoxia, and sepsis, appear to be a significant component of the pathogenesis of BPD [20, 32]. The immature immune system of preterm neonates leaded to aberrant regulation of lung inflammation [10, 33]. Therefore, we tested the relationship between cord blood LL37 and concentration of two main pro-inflammatory cytokines - IL-6 and TNF-a. Our study showed IL-6 level but not TNF-α was negatively associated with cord blood LL37. Damaged lung tissue releases chemotactic factors and inflammatory cytokines, such as interleukin IL-6 [33–35]. Multiple studies have shown that IL-6 was elevated very early in the respiratory course of the human preterm population that ultimately develop BPD [33–35]. Studies also indicated crosstalk between mesenchyme cells and alveolar epithelium cells may be disturbed by interleukin IL-6, therefore interrupt the pulmonary repairing process after injury [34]. The possible regulation of IL-6 by LL37 may be the underlying mechanism of LL37 for improving respiratory outcomes of very preterm infants. Further research on the mechanisms of LL37 regulation on IL-6 was needed.

## Limitations

There are several limitations of this study. Firstly, among the enrolled 112 very preterm infants less than 32 weeks, only 102 had available LL37 level results, 10 infants failed to test the LL37 level in cord blood. Therefore, when we analyzed the association between LL37 level and outcomes, only 102 infants with available LL37 results were included. As we used abandoned blood from routine blood tests 48–72 hours after birth for enzyme-linked immunosorbent assay test, and blood samples were collected only when it was clinically required during routine care. Thus, not all infants enrolled had available LL37 level results. Secondly, among 112 infants with a GA at birth < 32 weeks, only 14 infants had a GA at birth < 28 weeks. Further study should include more extremely preterm infants who are at greater risk of BPD and complications in general. Thirdly, elevated LL37 was found in respiratory infection condition, which is an acute inflammatory response process [15], however, BPD or COPD in adults are chronic lung inflammatory process. We found serum LL37 48–72 hours after birth had no association with BPD. Further study is needed to measure LL37 on different time points after birth and analyze the association of LL37 and chronic lung inflammation.

## Conclusion

The cord blood LL37 levels increased during gestation and decreased after perinatal steroid usage. Higher cord blood LL37 is an independent protective factor for developing BPD and severer BPD. The protective effect of LL37 on BPD and its severity may be mediated by an inhibiting function of pro-inflammatory cytokine IL-6. These results laid clinical foundation for the translational application of LL37 in preterm BPD.

## Supplementary Information

**Additional file 1:** **Supplemental Table 1.** Multiple regression analysis model for perinatal factors that may affect the concentration of LL37 in plasma 48–72 hours after birth. **Supplemental Table 2.** The main preterm complications in infants < 32 weeks and < 28 weeks.

## Data Availability

The datasets used and/or analyzed during the current study are available from the corresponding author on reasonable request.

## Abbreviations

IL-6: Interleukin-6
TNF-a: Tumor necrosis factor-a
BPD: Bronchopulmonary dysplasia
HBV: Hepatitis B virus
HCV: Hepatitis C virus
HIV: Human immunodeficiency virus
IgM: Immunoglobulin M
TORCH: Toxoplasma, rubella virus, cytomegalovirus, herpes simplex virus, and other
NICHD: Eunice Kennedy Shriver National Institute of Child Health and Human Development
NEC: Necrotizing enterocolitis
ROP: Retinopathy of preterm
ELISA: Enzyme-linked immunosorbent assay
IVH: Intraventricular hemorrhage
GA: Gestational age
