# Supplementary material for: Cord blood antimicrobial peptide LL37 levels in preterm neonates and association with preterm complications
Source: Ital J Pediatr. 2022 Jul 8;48:111. doi: 10.1186/s13052-022-01295-6 (PMC9270758; doi:10.1186/s13052-022-01295-6)
Supplement: Supplementary file 1 — Additional file 1: Supplemental Table 1. Multiple regression analysis model for perinatal factors that may affect the concentration of LL37 in plasma 48–72 hours after birth. Supplemental Table 2. The main preterm complications in infants < 32 weeks and < 28 weeks. [file 13052_2022_1295_MOESM1_ESM.docx]

| Variables | B | 95% CI for B | P |
| --- | --- | --- | --- |
| GA (weeks) | -13.767 | -82.232，54.696 | 0.689 |
| Male | -31.551 | -268.605，205.501 | 0.791 |
| Birth weight (kilogram) | 84.922 | -283.458，453.302 | 0.646 |
| Cesarean section delivery | 87.835 | -150.259，325.930 | 0.463 |
| Pregnancy-induced hypertension | -6.090 | -361.581，349.400 | 0.972 |
| GDM | 33.294 | -226.382，292.972 | 0.798 |
| Antenatal steroids usage | 11.959 | -266.268，290.187 | 0.931 |

Supplemental Table 1. Multiple regression analysis model for perinatal factors that may affect the concentration of LL37 in plasma 48-72 hours after birth.

B: unstandardized regression coefficient.

Dependent variable: LL37 in plasma 48-72 hours after birth.

CI: confidence interval;

GA: gestational age;

GDM: [gestational](C:/Users/renzh/AppData/Local/youdao/dict/Application/8.9.6.0/resultui/html/index.html#/javascript:;) [diabetes](C:/Users/renzh/AppData/Local/youdao/dict/Application/8.9.6.0/resultui/html/index.html#/javascript:;) [mellitus](C:/Users/renzh/AppData/Local/youdao/dict/Application/8.9.6.0/resultui/html/index.html#/javascript:;).

| Complications（n,%） | Infants <32 weeks(n=112) | Infants <28 weeks(n=14) |
| --- | --- | --- |
| BPD | 29 (25.9) | 8(57.1) |
| IVH | 43(38.4) | 5(35.7) |
| ROP | 18(16.1) | 6(42.9) |
| NEC | 21(18.8) | 2(14.3) |
| Sepsis | 14(12.5) | 6(42.9) |

Supplemental Table 2. The main preterm complication in infants <32 weeks and <28 weeks.

The median(range) for GA is 30.42(26.56-31.86) and birth weight is 1.355(0.55-2.32) in very 112 preterm neonates less than 32 GA. 112 infants had a GA at birth <32 weeks, and 14 infants had a GA at birth <28 weeks.

GA: gestational age; BPD-bronchopulmonary dysplasia; IVH-intraventricular hemorrhage; ROP-retinopathy of preterm; NEC-necrotizing enterocolitis.
